# Perceived face size in healthy adults

**DOI:** 10.1371/journal.pone.0177349

**Published:** 2017-05-08

**Authors:** Sarah D’Amour, Laurence R. Harris

**Affiliations:** Department of Psychology, Centre for Vision Research, York University, Toronto, ON, Canada; Macquarie University, AUSTRALIA

## Abstract

Perceptual body size distortions have traditionally been studied using subjective, qualitative measures that assess only one type of body representation–the conscious body image. Previous research on perceived body size has typically focused on measuring distortions of the entire body and has tended to overlook the face. Here, we present a novel psychophysical method for determining perceived body size that taps into implicit body representation. Using a two-alternative forced choice (2AFC), participants were sequentially shown two life-size images of their own face, viewed upright, upside down, or tilted 90°. In one interval, the width or length dimension was varied, while the other interval contained an undistorted image. Participants reported which image most closely matched their own face. An adaptive staircase adjusted the distorted image to hone in on the image that was equally likely to be judged as matching their perceived face as the accurate image. When viewed upright or upside down, face width was overestimated and length underestimated, whereas perception was accurate for the on-side views. These results provide the first psychophysically robust measurements of how accurately healthy participants perceive the size of their face, revealing distortions of the implicit body representation independent of the conscious body image.

## Introduction

It is crucial that our brain accurately represents our bodies in almost all situations, especially those involving perception and action. Yet, it is still not fully understood how we perceive the structure of our bodies or how the body is represented in the brain. Perceived body size is a fundamental construct that reflects our knowledge of self and is fundamental to all aspects of perception. It relies on mental body representation maps that have been assumed to be accurate in healthy adults. The motivation for the present study was to examine face size perception in healthy populations of males and females using a robust psychophysical method in order to measure implicit body representation rather than body image. While perceived face size may be correlated with body mass index (BMI) [1], here we reveal errors in people’s perception of their own face, showing that the assumption of an accurate body representation in healthy adults is not correct.

Research on body size perception has typically focused on the whole body or particular body parts that might be expected to have distortions, such as the stomach, thighs, or upper arms–areas of the body that tend to be higher in fat content [2–4]. However, since these studies were aimed at understanding distortions that occur in clinical populations, such as those with eating disorders, they have tended to ignore the face (but see [5]). Unlike many parts of the body, the face has some special attributes that make it unique—it has internal features, and it cannot be viewed directly: only in mirrors. Thus, visually guided interactions with the face (such as shaving or putting on makeup) are fundamentally different from interactions with our hands, arms, legs, etc. Face perception, including the ability to recognize and represent properties of the face, is a critical aspect of self-awareness that requires an accurate representation [6–10]. But how accurate is this representation? Previous research has shown that body image distortions occur in healthy people. Participants tend to overestimate the width of their shoulders and the length of their upper arms, while underestimating the lengths of their lower arms and legs. Interestingly, distortions were similar for both males and females [11]. Are such misrepresentations of the body reflected in the perceived size of the face?

Body size and shape perception have traditionally been investigated by exploring the conscious body image (a cognitive, subjective, mental body representation), most often in those with eating disorders [12–14]. Both perceptual and behavioral techniques have assessed body perception by using tasks that involve comparing a participant’s body with either pictures of their own body (depictive measures) or of objects (metric measures). These studies have revealed that people are inaccurate at judging their body size, suggesting that body perception distortions are found in both clinical and normal populations [2,14–22]. However, these studies have generally lacked rigorous experimental methods and the results have been mixed and inconsistent, depending on the factors that were used such as: experimental stimuli, methods, location, situation, concept definitions, etc. [3,14,23–26]. Studies that require picking a template or adjusting something to match perceived size involve the conscious body image. Such tasks require a subjective perspective that involves personal opinions and feelings that someone may have about their body size. This requires reflecting on their conscious beliefs about their size but doesn’t address the representation in the brain. Recent studies have explored body perception in normal populations using psychophysical techniques and have found that distortions exist even in healthy adults [27–29]. By using a two-alternative forced choice task (2AFC), we reduced response bias and the subjective nature of size judgments because participants no longer had to use a subjective opinion [30,31]. In our experiments, participants had to choose which of two photographs (an accurate reference photograph or a photograph that had been distorted in the horizontal or vertical dimension) was most like them. The end point was when the two images were judged as equally like them without either photograph necessarily matching their internal representation. The subjective element (in which a participant might choose, for example, a photograph that was most like how they would like to look) was thus removed by our method as there was no a priori reason to choose either photograph. This provided an implicit measure that taps into the brain’s body representation rather than an explicit measure that includes conscious and emotional aspects of body image. The implicit representation corresponds most closely to the representation in the brain and is not vulnerable to such emotional or cognitive influences. These judgments therefore provide a window into the representations in the brain that can be used for creating models and maps of how the brain perceives body size, shape, structure, etc. [5,11,16,32–39].

Here, we applied this technique to measure the perception of the face in a healthy population to determine how the face might be represented in the brain. We measured width and length accuracy in four different face orientations to obtain knowledge and baseline values of the distortion in the brain’s representation. We expected to find significant perceptual distortions, showing that distortions exist even in healthy populations. While we frequently see our face in the mirror, we don’t interact with it or see it in the same way that we do with the rest of our bodies. While we can see our hands directly in multiple different viewpoints, we only obtain visual information about our face from mirrors or photographs. This may lead to distortions of the representation of the face as the brain pieces together perceived size and shape from essentially a single perspective. We also predicted that there would be differences depending on the orientation in which the face was seen, with more pronounced errors when viewing the face in unfamiliar views. Previous literature has tended to test almost only female participants [13,40–42] and has shown that body distortions occur more in those dissatisfied with their bodies [43–48]. Here, we compared low and high body dissatisfaction groups in both male and female participants in order to add to the literature and advance knowledge about how gender and body satisfaction contribute to size perception. Based on the literature we expected that females and those with higher levels of body dissatisfaction would show greater perceptual distortions. We did not know how accurately males might perceive their face size.

## Methods

### Participants

**Fig 1:**
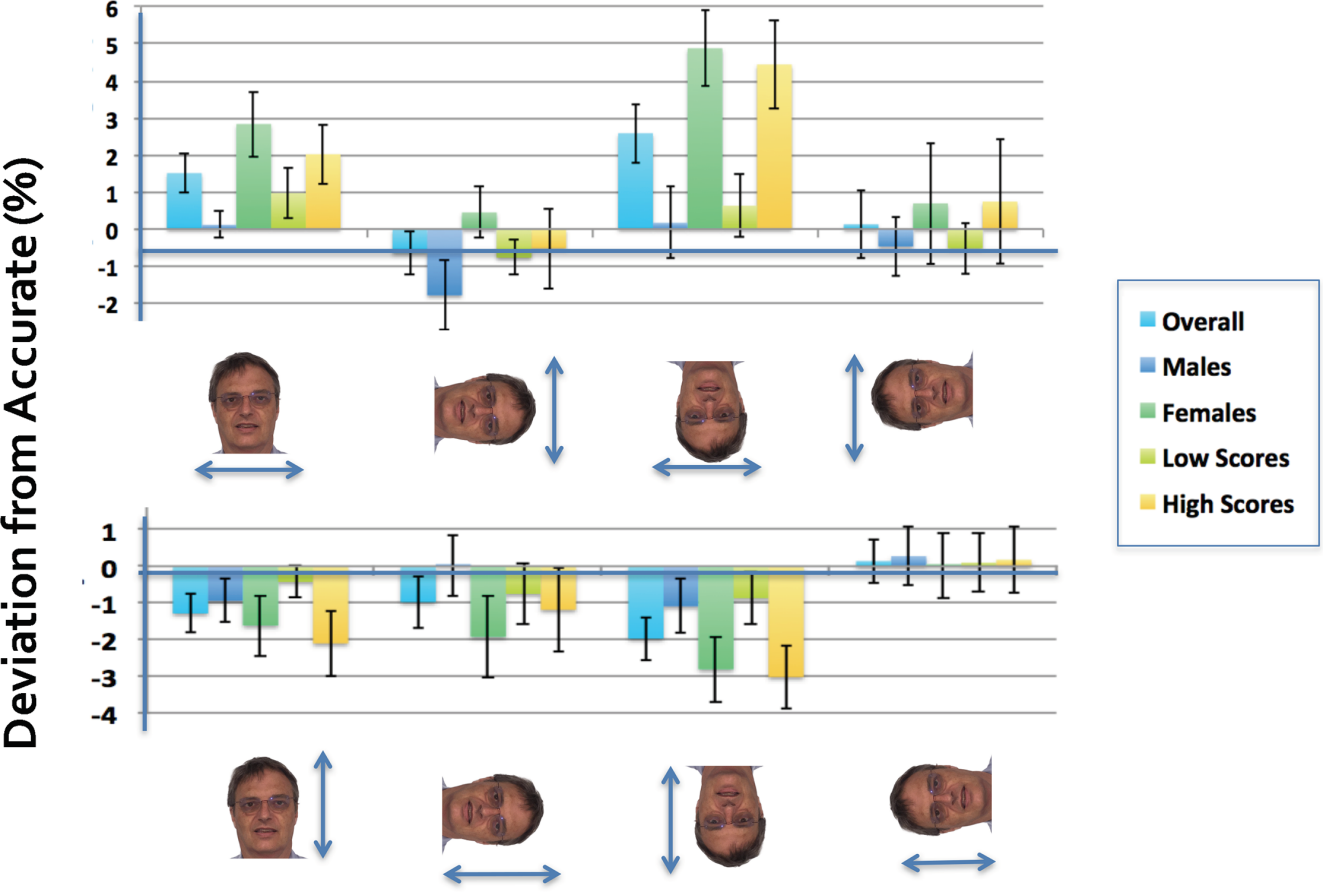
Deviation from accurate results when face width (top row) or face length (bottom row) was distorted for each viewing orientation. Average percentages are shown across all between-subjects groups. Positive and negative scores represent overestimation and underestimation respectively. Error bars represent ±1 SEM. The individual in this figure has given written informed consent (as outlined in PLOS consent form) to publish these case details.

Forty participants took part in the experiment (20 females, mean age 23.1 years, SD = 9.0 years). They were recruited from the York University Undergraduate Research Participant Pool and received course credit for taking part in the study. The experiment was approved by the York Ethics Board and all participants signed informed consent forms. The individual whose face is shown in Fig 1 in this manuscript has given written informed consent (as outlined in PLOS consent form) to publish these case details. The study was performed in accordance with the Treaty of Helsinki. The sample size was based on previous work to determine the power needed for significance and the potential variability within and between groups. Size for each gender group was kept equal, with an amount that would be sufficient to detect differences.

### Materials and stimuli

#### Body dissatisfaction

The Body Shape Questionnaire (BSQ) [49], a 34-item self-report questionnaire that was developed to assess concerns about body shape and experiences of feeling fat within the previous month, was administered before the experiment began to obtain a measure of body dissatisfaction. Higher scores indicate higher levels of body dissatisfaction. Participants were divided into high and low groups defined as whether their score was above or below the overall mean score.

#### Photographs

Color photographs of each participant were taken using an iPhone 6 camera (focal length = 4.15mm, sensor size = 4.8 x 3.6mm; 73° field of view). Participants were asked to stand in front of a wall with a neutral facial expression. Photographs were taken under standardized conditions with the flash on, from just above the head to the waist, from a distance of approximately 122cm with no zoom function. The image was then cropped and flipped to include only the participant’s head in mirror perspective, and formatted with a white background (Adobe Photoshop CS6). This image served as the undistorted “real” image and was used for composing the distorted images. Using a soft tape measure, face size was measured straight down from the top of the forehead to the bottom of the chin. The image was then adjusted to match the participant’s actual face size requiring a magnification 180x relative to the camera’s sensor size.

#### Distorting the images

Images were presented and distorted through the use of MATLAB (version 2011b) and Psychophysics Toolbox [50] running on a MacBook Pro. One dimension of the image (either width or length) was distorted (made either bigger or smaller) using a QUEST adaptive staircase psychometric procedure [51]. Optical distortions of the lens were confirmed as being less than 0.3% of the width or length of the photograph (given that the other length was set to veridical) using Matlab to assess the amount of distortion. The monitor was viewed orthogonally to the line of sight with the viewer aligned with in the center of the photograph. The image was displayed on the center of a computer monitor (27-inch Apple display) with the face in one of the four orientations shown in Fig 1: (1) upright (0°), (2) to the right (90°), (3) upside down (180°), and (4) to the left (270°). Width and length dimensions were distorted separately for each of the four orientations, in a block design resulting in eight conditions.

### Procedure

Participants sat in a chair approximately 45cm in front of the computer screen. Each trial consisted of two 1.5s intervals–one interval containing the undistorted image and one interval containing the distorted image—separated by a blank white screen. Participants identified which interval contained the image that most closely matched their own perception of their face and responded using a two-button computer mouse (left button for first interval, right button for second interval). A QUEST adaptive staircase procedure [51] was used with a two-alternative forced choice (2AFC) design to vary the chosen dimension (length or width) of the distorted image. Two QUEST staircases (25 trials per staircase) were used for each condition (50 trials total), with one starting with the manipulated dimension larger than natural and the other starting with that dimension smaller than natural. Each of the eight conditions was run in a single block and took approximately six minutes to complete. Block order was determined by a Latin square and was counterbalanced across participants.

It has been reported that viewing a photograph from a distance different from the camera’s focal length multiplied by the magnification (75cm in this case) can produce subtle perceptual effects [52]. We confirmed that viewing distance had no effect on our data by repeating our experiment on a selection of participants viewing the same photographs at a range of distances from 25–240cm.

### Data analysis

The QUEST program returned an estimate of the distortion needed for the participant to report that the distorted image was equally like their perceived face size as the undistorted image. The algorithm assumes the observer's psychometric function follows a Weibull distribution and adaptively determines the amount of distortion to be presented based on the participant's response to the previous trials. Knowledge of the observer's psychometric function accumulates throughout each experiment block. To visualize and confirm the QUEST’s performance, the participant’s decision was plotted against the distortion used for each trial and fitted with a logistic function (Equation 1) using the curve fitting toolbox in MATLAB.

$$\mathrm{Decision}=0.50+0.50/\left(1+\exp\left(\text{-}\left(x\text{-}x_{0}\right)/b\right)\right).$$ (1)

where x_0_ is the actual size, x is the distorted value that was equally likely to be judged as matching the observer’s face size and b is an estimate of the slope of the function. The perceived size of the face was taken as the point half way between x and x_0._

All of the 40 participants’ data for each condition was examined to validate the accuracy and efficiency of the QUEST procedure. The QUEST’s performance was also checked to determine whether reliable estimates were obtained within 50 trials. All data were inspected for outliers. Outliers were assessed as points that fell outside ± 3 standard deviations from the mean. Data that failed to meet these criteria were discarded from the analyses (one participant).

Estimates were converted to percentages relative to the accurate reference photograph (x*100/ x_0_) and data analyses were conducted on these values. One-sample *t* tests were conducted for each condition to assess whether accuracy errors differed significantly from zero *(p* values corrected for multiple comparisons with the false discovery rate method [53], alpha = .008). Mixed measures analyses of variances (ANOVAs) were used for statistical analyses, with alpha set at *p* < .05 and post-hoc multiple comparisons were made using Bonferroni corrections.

## Results

Table 1 summarizes the results of t-tests showing that the perceived width and length of upright and upside down faces were significantly different from accurate.

**Table 1:** One-sample t-tests comparing mean accuracy errors to 0.

		*M (SEM)*	*t*(38)	*p*	95% CI
Width					
	Upright	1.51(.53)**	2.87	.007	[.44, 2.57]
	Right	-0.64(.60)	-1.07	.290	[-1.85, .57]
	Upside Down	2.59(.80)**	3.24	.002	[.97, 4.20]
	Left	0.13(.92)	0.14	.890	[-1.73, 1.98]
Length					
	Upright	-1.28(.51)*	-2.50	.017	[-2.32, -.24]
	Right	-0.97(.71)	-1.38	.176	[-2.40, .46]
	Upside Down	-1.97(.59)*	-3.34	.002	[-3.16, -.77]
	Left	0.14(.60)	0.24	.814	[-1.07, 1.35]

Note: *N* = 39. CI = confidence interval.

*p < .025.

** *p* < .0125.

### Perceived face width

Fig 1 (upper graph) plots the deviation from accurate of width judgments for faces in each orientation tested (shown below the graph) with the participants divided into five categories: overall, males, females, low BSQ and high BSQ scorers. A three-way mixed ANOVA was conducted to test for within-subjects effects of orientation (upright, right, upside down, and left) and between-subjects effects of gender (male and female) and BSQ score (low and high groups). A significant main effect of orientation, *F*(3, 105) = 4.44, *p* = .006, *η*^*2*^_*ρ*_ = .113, was revealed, which implies that there were differences in perceiving face size depending on the orientation in which the image was viewed. Pairwise comparisons showed significant differences between the upright (*MD* = 2.26, *SE* = .61, *p* = .004) and upside down (*MD* = 3.16, *SE* = .86, *p* = .004) orientations compared to the right orientation. Participants overestimated face width by 1.44% ± .54 when the image was viewed upright and by 2.33% ± .76 when the face was upside down compared to being underestimated by 0.83% ± .64 in the right-side-down orientation. No significant interactions were found between orientation and gender, *F*(3, 105) = .92, *p* = .435, *η*^*2*^_*ρ*_ = .026, orientation and BSQ group, *F*(3, 105) = 1.06, *p* = .372, *η*^*2*^_*ρ*_ = .029, or between orientation, gender, and BSQ group, *F*(3, 105) = .82, *p* = .487, *η*^*2*^_*ρ*_ = .023.

There was a between-groups effect for gender, *F*(1, 35) = 5.67, *p* = .023, *η*^*2*^_*ρ*_ = .139, which indicates that males and females differ in perceived face width accuracy, but not for BSQ group, *F*(1, 35) = .39, *p* = .534, *η*^*2*^_*ρ*_ = .011. Females overestimated face width by 2.06% ± .73 whereas males underestimated by 0.39% ± .73 (*MD* = 2.46, *SE* = 1.03, *p* = .023). No significant interaction between gender and BSQ group, *F*(1, 35) = .01, *p* = .924, *η*^*2*^_*ρ*_ = .00, was found. To break down the effect of gender on orientation, a one-way ANOVA was conducted. Significant differences between males and females were only found for the upright, *F*(1, 37) = 7.89, *p* = .008, and upside down, *F*(1, 37) = 11.01, *p* = .002, orientations.

### Perceived face length

Fig 1 (lower graph) plots the deviation from accurate of length judgments for faces in each orientation with participants divided into the same five categories. A three-way mixed ANOVA was conducted to test for within-subjects effects of orientation (upright, right, upside down, and left) and between-subjects effects of gender (male and female) and BSQ score (low and high groups). A significant main effect of orientation, *F*(3, 105) = 2.83, *p* = .042, *η*^*2*^_*ρ*_ = .075, was observed but no significant interactions were found between orientation and gender, *F*(3, 105) = .70, *p* = .553, *η*^*2*^_*ρ*_ = .020, orientation and BSQ group, *F*(3, 105) = 1.16, *p* = .329, *η*^*2*^_*ρ*_ = .032, or between orientation, gender, and BSQ group, *F*(3, 105) = .89, *p* = .446, *η*^*2*^_*ρ*_ = .025. Pairwise comparisons did not show any significant differences between the orientations.

There was no between-groups effect for gender, *F*(1, 35) = .95, *p* = .336, *η*^*2*^_*ρ*_ = .027, or for BSQ group, *F*(1, 35) = .62, *p* = .436, *η*^*2*^_*ρ*_ = .017. However, a significant interaction between gender and BSQ group, *F*(1, 35) = 5.29, *p* = .028, *η*^*2*^_*ρ*_ = .131, was found. To follow up on this interaction, simple effects tests were conducted. Face size accuracy errors significantly differed between males and females for the high BSQ group (*MD* = 2.85, *SE* = 1.22, *p* = .026) but not for the low scoring group (*MD* = 1.15, *SE* = 1.24, *p* = .359). Females in the high BSQ group underestimated the length of their face by 2.38% ± .67 compared to those in the low BSQ group who overestimated by .31% ± 1.02 (*MD* = 2.69, *SE* = 1.22, *p* = .035) whereas males in the low and high BSQ groups did not differ.

## Discussion

We have measured how accurately healthy participants perceive the size of their face in four different orientations. This is the first time that this has been done using psychophysically robust measurements. We have showed that neither healthy males nor healthy females are completely accurate at judging the length and width of their faces, and that accuracy changes depending on the orientation in which the face is viewed. Curiously, the largest errors were found in the most familiar orientations, whereas participants were accurate when the face was viewed in the unfamiliar sideways orientation.

### Overall accuracy

Our finding that people perceive their face as wider and shorter than it actually is, regardless of body dissatisfaction or gender, is a novel finding that brings the traditional belief–that perceived body distortions are unique to females who are dissatisfied with their bodies [3,23,26,43,44]—into question. It has been assumed that healthy people have accurate body representations and only very recently has research begun to challenge this assumption [5,11,32,33,35,54–56]. While these studies have started to explore body size perception, only a few studies have looked specifically at the face or head. Perceived length of body parts relative to each other was measured, finding that head-length estimates were mixed but mostly overestimated [5]. This finding is not compatible with our results of underestimated face length. However, our findings are supported by several studies that found similar distortion in body representation. For example, Fuentes et al. [57] investigated facial feature representation and showed an overall bias towards a short and wide face representation, and comparable distortions have also been observed for the hands [33–35,58,59] and overall body shape [16].

### Gender and body satisfaction scores

Here, we show that healthy people of both genders demonstrate distortions in perceived face size, with males and females differing only in the magnitude of their errors. While a link between body satisfaction and perceived body size distortions is known [60–63] previous research has typically focused on comparing healthy and eating disorder groups, which are known to have clinical levels of body dissatisfaction. From these earlier studies, it has been assumed that those with higher BSQ scores, even within the healthy range, would show greater amounts of distortion compared to those with lower scores. Surprisingly, we found that BSQ scores had an effect only on perceived face length: females in the high BSQ group underestimated face length compared to those in the low BSQ group, whereas males did not. Finding no effect of BSQ group on perceived face width tells us that this perceptual distortion depends on more than gender and body dissatisfaction levels.

### The effect of orientation

We expected that accuracy would worsen when the face was presented in unfamiliar views. That is, participants would be most accurate when viewing their face in the upright orientation, less accurate in left and right orientations, and worst when the face was presented upside down. However, our results did not match this pattern and we found that instead perceptual distortions only occurred when the face was presented upright or upside down and that participants were accurate (scores not different from 0) when the face was viewed tilted to the left or right. It is not clear why this might be. One possibility is that there is something special about the face when it is in typical face form–upright or upside down are both in the regular face shape orientation–whereas when the face is tilted to the left or right, it is taken out of the typical form and becomes a distinctive shape that is never normally seen. Creating such a peculiar view might cause a person to feel that the face is no longer theirs and they come to perceive the image as an external neutral object unrelated to the self. Thompson and Wilson [64] showed that an inverted face appeared thinner than an upright face and that the effect appeared to be driven by the internal features of the face. They removed the distortion by removing the internal features. It seems that placing the features in an unusual orientation (by turning the face sideways) has the same effect, restoring accurate face size perception in all males and females.

## Conclusions

The main objective of this study was to assess accuracy of the perceived face dimensions to develop a method of assessing body representation. We have shown, for the first time, how the brain quantitatively perceives the size of the face by measuring baseline values of perceived distortions with the face in several orientations. The implicit representation of the face in the brain does not appear to be the same as the face seen in the mirror. These data reveal how accurately people are able to judge their body dimensions and provide insight into how body size and shape are processed and represented by the brain. Future research will use this method to further examine the perception of body dimensions to quantify and compare how distortions vary under different experimental designs, such as testing the effects of image size, different body parts, and the whole body in order to develop a comprehensive understanding of the three-dimensional representation of the body in the brain.

## Supporting information

S1 File Data. The percentage difference from accurate where 0 = accurate and negative means thinner or shorter than accurate.
(XLSX)
